# Automated Insulin Delivery for Patients with Type 2 Diabetes: A Narrative Review

**DOI:** 10.2174/0115733998418695251203105337

**Published:** 2026-03-02

**Authors:** Konstantinos Kitsios, Christina-Maria Trakatelli

**Affiliations:** 1 Third Department of Internal Medicine, Aristotle University of Thessaloniki, Papageorgiou General Hospital, Thessaloniki, Greece

**Keywords:** Artificial pancreas, automated insulin delivery, fully closed-loop system, hybrid closed-loop system, hypoglycemia, type 2 diabetes

## Abstract

Despite the availability of newer antidiabetic medications, many patients with type 2 diabetes mellitus still do not achieve their glycemic targets and require insulin treatment. Research from a limited number of randomized controlled trials and observational studies suggests that Automated Insulin Delivery (AID) systems are safe and effective for treating individuals with type 2 diabetes. With the implementation of a hybrid insulin pump, an average reduction in HbA1c of 0.6 to 0.9 percentage points is achieved, along with an increase in Time in Glucose Range (70-180 mg/dL) (3.9-10.0 mmol/L) of 7 to 21 percentage points. This improvement corresponds to an additional 1.7 to 5.1 hours of euglycemia per day, without increasing the risk of hypoglycemia or diabetic ketoacidosis, and with a slight increase in body weight, potentially reflecting improved glycemic control. Concomitant treatment with GLP-1RA and/or SGLT-2 inhibitors has minimal effect on the magnitude of glycemic improvement achieved after switching to AID therapy, although evidence remains limited. Both the effectiveness and device-related adverse reactions are similar to those observed in patients with type 1 diabetes. Although individuals with type 2 diabetes who require insulin treatment are generally older and may be less familiar with technology, training in the necessary skills and adherence to AID use in real-world settings appear feasible. Treatment with AID may slightly improve quality of life and reduce diabetes-related stress.

## 1 INTRODUCTION

Automated insulin delivery (AID) in the form of a hybrid insulin pump significantly improves glycemic control in patients with type 1 diabetes mellitus (T1DM) [1-3]. Despite the emergence of new classes of antidiabetic medications, a notable number of patients with type 2 diabetes mellitus (T2DM) still fail to achieve their glycemic targets and may require insulin therapy [4]. Patients using multiple daily injections (MDI) or insulin pumps could also benefit from hybrid AID systems; however, safety and efficacy data in T2DM remain limited and are derived mainly from hospitalized patients or small pilot studies conducted under controlled conditions [5-10]. Search strategy included PubMed using the following search string: ((closed-loop) OR (hybrid closed-loop)) OR (closed loop)) OR (hybrid closed loop)) OR (automated insulin delivery)) OR (omnipod)) OR (omnipod5)) OR (omnipod 5)) OR (Diabeloop)) OR (DBLHU)) OR (DBLG1)) OR (CamAPS Fx)) OR (control IQ)) OR (control-IQ)) OR (controlIQ)) OR (670G)) OR (Minimed 670g)) OR (780G)) OR (Minimed 780g)) AND ((Diabetes Mellitus) OR (diabet*)) and CENTRAL using the following search string: (diabet*) AND (Diabetes Mellitus) AND (Minimed 780g):ti,ab,kw OR (780G):ti,ab,kw OR (Minimed 670g):ti,ab,kw OR (670G):ti,ab,kw OR (controlIQ):ti,ab,kw OR (control-IQ):ti,ab,kw OR (control IQ):ti,ab,kw OR (CamAPSFx):ti,ab,kw OR (DBLG1):ti,ab,kw OR (DBLHU):ti,ab,kw OR (Diabeloop):ti,ab,kw OR (omnipod 5):ti,ab,kw OR (omnipod5):ti,ab,kw OR (omnipod):ti,ab,kw OR (automated insulin delivery):ti,ab,kw OR (hybrid closed loop):ti,ab,kw OR (closed loop):ti,ab,kw OR (hybrid closed-loop):ti,ab,kw OR (closed-loop):ti,ab,kw for relevant published trials from inception until September 26^th^, 2025. This review aims to critically evaluate the existing literature, focusing primarily on results from randomized clinical trials in real-world settings.

## 2 EVIDENCE FROM RANDOMIZED CLINICAL TRIALS

In the largest clinical trial conducted to date, 319 patients with a mean age of 59 years and a mean duration of T2DM of 18 years were randomly assigned in a 2:1 ratio to receive an AID system (t:slim X2 insulin pump with Control-IQ+ technology, Tandem, in conjunction with a Dexcom G6 sensor) or to continue their existing treatment regimen. The control group maintained their current approach, which involved multiple daily injections of both basal and mealtime insulin or the use of an insulin pump, alongside continuous real-time glucose monitoring [11].

The primary endpoint was the change in glycosylated hemoglobin (HbA1c). After 13 weeks of intervention, the AID group demonstrated a significant reduction in HbA1c, decreasing from 8.2 ± 1.4% (66.1 ± 15.3 mmol/mol) to 7.3 ± 0.9% (56.3 ± 9.8 mmol/mol), representing a drop of 0.9 percentage points (9.8 mmol/mol). In contrast, the control group experienced a more modest reduction of 0.3 percentage points (3.3 mmol/mol), from 8.1 ± 1.2% (65 ± 13.1 mmol/mol) to 7.8 ± 1.1% (61.7 ± 12.1 mmol/mol). The mean adjusted difference between the two groups was -0.6 percentage points (-6.5 mmol/mol), with a 95% confidence interval (CI) of -0.8 to -0.4 (-8.7 to -4.4 mmol/mol) (*p* < 0.001).

Additionally, the percentage of time that glucose levels were maintained within the therapeutic Target in Range (TIR) of 70-180 mg/dL (3.9-10.0 mmol/L) increased from 48 ± 24% to 64 ± 16% in the AID group, while the control group showed only a slight change from 51 ± 21% to 52 ± 21%. The mean difference in TIR between the two groups was 14 percentage points, with a 95% CI of 11 to 17 (*p* < 0.001). Notably, the occurrence of hypoglycemia was very low in both groups, with only one episode of severe hypoglycemia reported in a patient using the AID system.

Importantly, the mean total daily insulin dose decreased from 95 ± 47 units at baseline to 87 ± 46 units at 13 weeks in the AID group, whereas it increased from 102 ± 50 units to 104 ± 56 units in the control group. The mean change from baseline in weight was 2.4 ± 4.4 kg in the AID group and 0.9 ± 3.3 kg in the control group. Although the mechanism underlying the slight weight gain observed in the context of reduced total daily insulin delivery remains unclear, the investigators proposed decreased glycosuria-particularly in patients with higher baseline HbA1c levels-or increased calorie intake as possible explanations.

Most of the Patient-Reported Outcomes Surveys (PROs) did not differ between treatment groups. In a subgroup analysis, the treatment effect of AID did not differ between participants receiving Glucagon-Like Peptide-1 Receptor Agonists (GLP-1RA) or Sodium-Glucose Co-Transporter 2 inhibitors (SGLT-2inh) and those who were not taking these medications. However, patients treated with both SGLT-2 inhibitors and GLP-1RA exhibited a greater reduction in HbA1c compared to those not receiving these agents.

During a crossover clinical trial involving 17 patients with a mean age of 63 years, a mean HbA1c of 7.9% (62.8 mmol/mol), and a mean duration of type 2 diabetes of 18 years, participants on insulin pump therapy and Continuous Glucose Monitoring (CGM) were randomized to receive either a hybrid pump (DBLG1) or insulin pump therapy with CGM in an open-loop system over two 6-week periods [12]. The primary endpoint was Time in Range (TIR): 70-180 mg/dL (3.9-10.0 mmol/L). TIR increased to 76% with the closed-loop system compared with 61% in the open-loop phase (mean difference 15 percentage points, interquartile range [IQR] 8.0-22.0; *p* < 0.001).

The hybrid pump significantly reduced time spent in hyperglycemia, glucose coefficient of variation (CV), and glucose management index (GMI). Time in hypoglycemia was low and did not differ between the two interventions. In contrast, the daily insulin dose was significantly higher with the hybrid pump.

There was no change in weight from the beginning to the end of the study for either the entire population or for each intervention group separately. The mean sleep fragmentation index was lower with the closed-loop system than with conventional treatment (P = 0.047). However, the remaining PROs did not differ between interventions.

In a parallel-design randomized trial, the Tandem t:slim X2 Control-IQ technology hybrid insulin pump (Tandem Diabetes Care) was evaluated against multiple daily insulin injections (MDI) in a cohort of 30 patients with T2DM who had a mean age of 69 years and required residential care [13]. The primary endpoint was TIR: 70-180 mg/dL (3.9-10.0 mmol/L), while secondary endpoints included other parameters of the ambulatory glucose profile, HbA1c, and daily insulin doses. After 12 weeks of treatment, compared with MDI, the use of AID significantly increased TIR from 34.4% to 63% (adjusted mean difference 27.4%; 95% CI 15.0-39.8; *p* < 0.001), with no significant difference in time spent in hypoglycemia. In the AID group, HbA1c levels also showed a significant reduction, decreasing from 9% (74.9 mmol/mol) to 7.4% (57.4 mmol/mol) (adjusted mean difference -1.3%, 95% CI -1.9 to -0.7; −14.2 mmol/mol, 95% CI -20.8 to −7.7; *p* < 0.001).

There were no episodes of severe hypoglycemia or diabetic ketoacidosis (DKA) in either intervention group. Insulin use increased by 36.3 ± 65.5 U/day in the control group and by 13.8 ± 75.6 U/day in the intervention group (P = 0.52). Weight decreased by 0.76 ± 4.17 kg in the control group and increased by 1.62 ± 2.88 kg in the intervention group (*p* = 0.08). However, none of these differences were statistically significant. Satisfaction with hybrid pump therapy was high among both patients and caregivers, and by the end of the study, 77.8% of patients were willing to continue using the AID system.

The Cambridge group investigated the safety and efficacy of a fully automated closed-loop insulin delivery system through two crossover clinical trials. In the first trial, 26 adults with T2DM requiring dialysis, with a mean age of 68 years and a mean duration of diabetes of 20 years, were randomly assigned to two 20-day treatment periods using the CamAPS HX fully closed-loop app with ultra-rapid insulin aspart or conventional insulin therapy paired with a blinded CGM [14]. The primary endpoint was TIR: 70-180 mg/dL (3.9-10.0 mmol/L). TIR achieved with closed-loop insulin delivery was 52.8 ± 12.5%, compared with 37.7 ± 20.5% using conventional therapy, resulting in a mean difference of 15.1 percentage points (95% CI 8.0-22.2; *p* < 0.001).

Time spent in hypoglycemia (<70 mg/dL; 3.9 mmol/L) was significantly lower with the closed-loop system (0.1%, IQR 0.0-0.4%) compared with conventional therapy (0.2%, IQR 0.0-0.9%) (*p* = 0.04). Notably, one episode of severe hypoglycemia occurred during the closed-loop period, but it took place when the algorithm was not operational. Total daily insulin dose did not differ between treatments. Most participants reported spending less time managing their diabetes when using the closed-loop system and indicated they would recommend it to others.

In a second trial, 28 adults with T2DM, with a mean age of 59 ± 11 years and a mean HbA1c of 75 ± 15 mmol/mol (9.0% ± 1.4%), were randomly assigned to two 8-week treatment periods using the fully automated closed-loop insulin delivery system (CamAPS HX app) or conventional insulin therapy combined with a blinded CGM [15]. The primary endpoint was TIR: 70-180 mg/dL (3.9-10.0 mmol/L). Closed-loop therapy achieved a TIR of 66.3 ± 14.9%, compared with 32.3 ± 24.7% during conventional insulin therapy, yielding a mean difference of 35.3 percentage points (95% CI 28.0-42.6; *p* < 0.001).

Closed-loop therapy also resulted in significantly lower HbA1c values than conventional therapy, with levels of 57 ± 9 mmol/mol (7.3% ± 0.8%) versus 72 ± 13 mmol/mol (8.7% ± 1.2%), respectively. This corresponds to a mean difference of -15 mmol/mol (95% CI -11 to -20 mmol/mol), equivalent to -1.4% (95% CI -1.0 to -1.8%; *p* < 0.001).

Additionally, time spent in hypoglycemia (<70 mg/dL; 3.9 mmol/L) showed no significant difference between the two interventions, and no episodes of severe hypoglycemia were reported. There was only one serious treatment-related adverse event, an infusion-site abscess, during the closed-loop therapy period. Total daily insulin dose was higher during closed-loop therapy (median 0.90 U/kg/day; IQR 0.72-1.63 U/kg/day) compared with the control therapy period (median 0.71 U/kg/day; IQR 0.56-1.26 U/kg/day).

Hypoglycemia confidence scores and Problem Areas in Diabetes (PAID) scores were similar between interventions, while hypoglycemia worry scores were higher during closed-loop therapy. The baseline characteristics of participants included in the randomized controlled trials are summarized in Table **1**. Tables **2A** and **B** summarize the outcomes of the randomized controlled trials.

In a recent meta-analysis of seven randomized controlled trials in patients with T2DM, a fully closed AID system, when compared with conventional insulin treatment, demonstrated a significant increase in time spent in euglycemia both overnight and during the day, with no difference in time spent in hypoglycemia [16]. However, five of the trials included in this meta-analysis were conducted in hospitalized patients [5-9].

## 3 NON-RANDOMIZED AND REAL-WORLD STUDIES

The safety and efficacy of AID systems in patients with T2DM have been investigated in a limited number of uncontrolled pilot and real-world studies. In the study by Davis *et al.*, 24 patients with T2DM-mean age 61 years, mean HbA1c 9.4% (79.2 mmol/mol), and an average disease duration of 19 years-who were using basal insulin or an MDI regimen, with or without CGM, were transitioned to AID therapy with the Omnipod 5 hybrid system [17]. After 8 weeks of treatment, the time spent with glucose levels exceeding 250 mg/dL (13.9 mmol/L) decreased by 16.9 percentage points (*p* < 0.0001). This improvement was accompanied by a significant increase in TIR of 21.9 percentage points (*p* < 0.0001), while maintaining a low incidence of hypoglycemia, defined as glucose levels below 54 mg/dL (3.0 mmol/L). Additionally, HbA1c was reduced by 1.3 percentage points (14.2 mmol/mol) (*p* < 0.0001), with no significant changes in body weight. No episodes of severe hypoglycemia or DKA were recorded. Among participants previously treated with MDI, total daily insulin decreased by 32% (*p* = 0.003). Psychosocial outcomes assessed using validated questionnaires significantly favored AID treatment.

Twenty-two participants continued AID therapy during a 26-week extension phase, achieving a further decrease in time spent with glucose levels above 250 mg/dL (13.9 mmol/L) to 9.7% ± 9.2% [18]. The percentage of time spent < 54 mg/dL (3.0 mmol/L) remained low, while HbA1c decreased by 1.6% ± 1.2% (15.5 ± 13.1 mmol/mol, *p* < 0.0001), and TIR increased by 22.4% ± 19.2% (*p* < 0.0001) from standard therapy through the extension period. These improvements occurred without significant changes in body mass index and without an increase in total daily insulin requirements.

A recent study examined the safety and efficacy of the Omnipod 5 system in 305 patients with T2DM, with an average age of 57 years. These individuals were treated with various insulin regimens, including basal insulin alone, MDI, or an insulin pump, with or without CGM. Additionally, some participants used other antidiabetic agents such as GLP-1RA and SGLT-2 inhibitors [19]. After 13 weeks of AID treatment, HbA1c decreased from 8.2% (66.1 mmol/mol) to 7.4% (57.4 mmol/mol) (mean difference −0.8 percentage points [95% CI, −1.0 to −0.^7^], equivalent to −8.7 mmol/mol [95% CI, −10.9 to −7.^6^]; *p* < 0.001 for both noninferiority and superiority). TIR (70-180 mg/dL) (3.9-10.0 mmol/L) increased from 45% to 66% (mean difference 20 percentage points [95% CI, 18 to 22], *p* < 0.001). The proportion of time spent in hypoglycemia <70 mg/dL (3.9 mmol/L) and <54 mg/dL (3.0 mmol/L) did not differ significantly with AID therapy, although one episode of severe hypoglycemia was reported. The reduction in HbA1c was consistent regardless of concomitant treatment with SGLT-2 inhibitors or GLP-1RA. Body weight increased by 0.8 kg (*p* < 0.001), while total daily insulin significantly decreased from a mean (SD) of 0.80 (0.46) U/kg/day at baseline to 0.57 (0.29) U/kg/day with AID (*p* < 0.001). Most patient-reported outcomes assessed in the study favored AID treatment.

Bhargava *et al.* conducted a study to evaluate the safety and efficacy of the MiniMed 780G hybrid system in 95 patients with T2DM, who had a mean age of 60.3 years and a mean disease duration of 18.6 years [20]. These patients were treated with either MDI or an insulin pump, with or without CGM. After three months of treatment with the hybrid closed-loop insulin delivery system, HbA1c decreased from 7.9% (62.8 mmol/mol) to 7.2% (55.2 mmol/mol) (*p* < 0.001). TIR (70-180 mg/dL) (3.9-10.0 mmol/L) improved significantly, rising from 72.2% to 79.8% (mean difference 7.2%, *p* < 0.0001), while the time spent in level 1 and level 2 hypoglycemia remained stable. Total daily insulin requirements increased from 77.4 to 91.8 units (mean difference 13.9 units, *p* < 0.0001), with no change in body weight. Notably, no cases of severe hypoglycemia, DKA, or hyperosmolar hyperglycemic state were recorded. Psychometric questionnaire scores improved with AID use, including reductions in overall Diabetes Treatment Satisfaction Questionnaire (DTSQ) scores and in perceived frequencies of hyperglycemia and hypoglycemia, indicating enhanced quality of life.

In a real-world study, Breton and Kovatsev collected data over one year from 378 patients with T2DM using a hybrid insulin delivery system featuring the Control-IQ algorithm [21]. Compared with the previous treatment regimen, TIR significantly increased with the AID system, rising from 69.9% to 78.0% (*p* < 0.001). This improvement was accompanied by a reduction in the duration of hyperglycemia in the 180-250 mg/dL (10.0-13.9 mmol/L) range, which decreased from 23.9% to 19%, and a reduction in time spent with glucose levels exceeding 250 mg/dL (13.9 mmol/L), which fell from 3.6% to 2.3% (*p* < 0.001). Additionally, time in hypoglycemia below 54 mg/dL (3.0 mmol/L) increased slightly, from 0.00% to 0.04% (*p* < 0.001). However, time in hypoglycemia within the 54-70 mg/dL (3.0-3.9 mmol/L) range remained unchanged at 0.2%.

Table **3** summarizes the baseline characteristics of participants in non-randomized and real-world studies. The reported outcomes of these studies are presented in Table **4**.

## 4 DISCUSSION

Automated insulin delivery systems for treating insulin-treated patients with T2DM appear to be safe and effective. With the implementation of a hybrid insulin pump, an average reduction in HbA1c of 0.6 to 0.9 percentage points is achieved, along with an increase in TIR (70-180 mg/dL) of 7 to 21 percentage points, equivalent to 1.7 to 5.1 additional hours of euglycemia per day, without an increase in time spent in level 1 or level 2 hypoglycemia. Small absolute increases in time <54 mg/dL, although statistically significant, are clinically negligible and should be interpreted accordingly [21]. The effectiveness of AID treatment in T2DM patients is comparable to results observed in randomized clinical trials involving patients with T1DM [22, 23]. Furthermore, the improvement in glycemic control is achieved safely within the first weeks of treatment, without an increased risk of severe hypoglycemia or DKA. Adverse reactions associated with the devices and infusion systems are also comparable to those seen in patients with T1DM [24, 25].

Interestingly, the improvement in glycemic control appears to be greater in patients with HbA1c >9% (74.9 mmol/mol) when switching to AID [11, 17, 19]. In some studies, a slight increase in body weight has been observed in these patients despite a reduction in daily insulin requirements. This minimal increase may reflect normalization of glucosuria, especially among patients with higher baseline glycated hemoglobin levels, or increased caloric intake [11]. However, attributing this change to “improved control” is speculative, and more studies are needed to address concerns regarding clinically significant weight gain with AID use in patients with T2DM.

Following treatment guidelines, an increasing number of patients with T2DM and a history of cardiovascular disease are being treated with GLP-1RA and/or SGLT-2 inhibitors [26]. However, almost half of the T2DM participants in AID trials were not treated with these classes of cardioprotective anti-diabetic medications. In a randomized placebo-controlled trial in patients with T1DM, semaglutide as an adjunct to AID treatment resulted in significant increases in TIR and decreases in weight and daily insulin needs without increasing time spent in hypoglycemia [27]. In an observational prospective study, tirzepatide, a dual GLP-1/GIP agonist, used as adjunct therapy to AID reduced total daily insulin requirements and weight while increasing TIR in patients with T1DM [28]. In T2DM, concomitant use of GLP-1 RAs or SGLT2i has shown minimal additional benefit to AID efficacy. However, these findings are derived from subgroup analyses with potentially limited power to detect existing differences and therefore warrant further investigation in larger controlled studies [11, 19].

The effectiveness of hybrid pump therapy appears unaffected by prior treatment regimen or CGM use [11, 19]. Although patients with T2DM on insulin are generally older and may be less familiar with technology, structured training enables successful AID implementation in real-world practice [29-34]. Moreover, precise carbohydrate counting does not seem necessary for effective AID use in T2DM, as most trials relied on qualitative meal-size assessments [11, 19].

It is noteworthy that, unlike patients with T1DM-where the increase in TIR during nighttime is significantly greater than during the day-in patients with T2DM, the increase in euglycemia occurs equally during both night hours and daytime hours [35-37]. Patients generally comply well with the treatment, remaining in the system’s auto-mode for more than 90% of the total time.

Studies of AID therapies in patients with T1DM suggest increased treatment satisfaction, improved quality of life, and decreased diabetes-related distress, likely driven by the prevention of hypoglycemia [38-43]. Evidence regarding the impact of diabetes technologies on patient-reported outcome measures (PROMs) in individuals with T2DM remains limited [44]. Available data are mixed: while most studies report improved satisfaction and reduced treatment burden, some document persistent or increased hypoglycemia-related worry [15, 19, 21, 45-47]. This heterogeneity in PROs should be acknowledged, as it highlights the need for further research to better understand patient-specific factors influencing these outcomes.

In this review, we explored the efficacy and safety of AID treatment in patients with T2DM. We focused on clinical trials that enrolled patients already treated with insulin alone or in combination with other oral or injectable antidiabetic agents under real-life conditions. However, many relevant questions remain unanswered. Our findings do not apply to patients with T2DM who are not treated with insulin, as initiation of insulin therapy using AID has not yet been studied. Some trials enrolled special populations, such as patients on dialysis or those requiring residential assistance, and therefore their results may not be generalizable to the broader T2DM population. Adjunctive GLP-1RA or SGLT2i therapy may theoretically enhance outcomes such as weight reduction or lower insulin requirements, but supporting data in T2DM remain insufficient and warrant further investigation.

The cost of AID treatment is high and may pose a barrier to implementation in individuals with T2DM. Additionally, there is a lack of data regarding the cost-effectiveness of AID use in this population. Finally, a comprehensive analysis of the broader impact of diabetes technology on the treatment of patients with T2DM was beyond the scope of this review.

## CONCLUSION

AID is an effective and safe treatment option for insulin-treated patients with T2DM who do not achieve their glycemic goals. It significantly decreases HbA1c and increases TIR, while time spent in hypoglycemia remains unchanged. The improvement in glycemic control is accompanied by a slight increase in weight and a modest improvement in quality of life. The need for training in specific technical skills and the associated cost may be potential barriers to AID treatment for many individuals with T2DM. Larger, long-term randomized clinical trials are needed to define the optimal application and cost-effectiveness of various AID algorithms in broader T2DM populations, as well as to identify which patient subgroups stand to benefit most from this technology.

## Figures and Tables

**Table 1:** Baseline characteristics of the participants in randomized controlled trials.

**Study ID**	**Design**	**AID**	**Comparator**	**Duration**	**Sample size (N)**	**Age (yrs)**	**Diabetes duration (yrs)**	**BMI** **(Kg/m^2^)**	**HbA1c** **(mmol/mol))**	**TIR** **(%)**	**Insulin dose(u/d)**	**GLP-1RA N(%)**	**SGLT-2 inh** **N(%)**
Kudva *et al. *[11]	RCT parallel	Control-IQ	MDI+CGM	13 weeks	319	59(12)	18 (11-26)	33 (29-40)	66.1(15.3)	48 (24)	95(47)	141 (44)	117 (37)
Borel *et al. *[12]	RCT cross-over	DBLG1	CSII + CGM	6 weeks	17	63(9)	24(9)	32(4)	62.8(9.9)	58.9 (20.1)	78.1(35.9)	7 (41)	5 (29)
Reznik *et al. *[13]	RCT parallel	Control-IQ	MDI	12 weeks	29	69.3(6.7)	20.4 (12.3)	32.3 (4.3)	74.9(13.1)	34.4 (21.3)	62.8(37.2)	10 (34)	0
Boughton *et al. *[14]	RCT cross- over	CamAPS HX fully closed-loop	Standard insulin therapy	20 days	26	68.3(11.2)	20.0 (10.0)	30.4 (6.5)	55.2 (14.2))	37.7 (20.5)	32.2(12.1-54.4)	0	0
Daly *et al. *[15]	RCT cross-over	CamAPS HX fully closed-loop	Standard insulin therapy + masked CGM	8 weeks	26	59 (11)	17.5 (8.2)	35.3 (8.6)	74.9(15.3)	32.3 (24.7)	84 (54-129)	11 (42)	6 (23)

Data are reported as mean (SD), or median (IQR) unless otherwise indicated. AID: Automated Insulin Delivery. MDI: Multiple Daily Insulin injections. CSII: Continuous Subcutaneous Insulin Infusion. CGM: Continuous Glucose Monitoring. BMI: Body Mass Index. TIR: Time In Range. GLP-1RA: Glucagon-Like Peptide 1 Receptor Agonist. SGLT-2inh: Sodium-Glucose Co-Transporter 2 inhibitor. RCT: Randomized Controlled Trial.

**Table 2A:** Treatment outcomes of the participants in randomized controlled trials.

**Study ID**	**BMI** **(Kg/m^2^)**	**BMI-EOT (Kg/m^2^)**	**BMI-control (Kg/m^2^)**	**BMI-control EOT (Kg/m^2^)**	**ΔBMI** **(95% CI) (Kg/m^2^)**	***p***	**HbA1c** **(mmol/mol))**	**HbA1c-ΕΟΤ (mmol/mol)**	**HbA1c-control (mmol/mol)**	**HbA1c-control EOT(mmol/mol)**	**ΔHbA1c (95% CI)**	***p***
Kudva *et al. *[11]	34.2(7.1)	35.1(7.5)	35.2(7.7)	35.5(7.6)	0.6(0.2 to 1.0)	NA	66.1(15.3)	56.3 (9.8)	65(13.1)	60.7(12)	-6.5(-8.7 to -4.4)	<0.001
Borel *et al. *[12]	32(4)	NA*	32(4)	NA*	NA*	NA*	62.8(9.9)	NA	NA	NA	NA	NA
Reznik *et al. *[13]	32.3 (4.3)	NA	35.6(6.5)	NA	NA**	NA	74.9(13.1)	57.4(6.5)	77.6(10.9)	72.7(10.9)	-14.2(-20.8 to -7.7)	<0.001
Boughton *et al. *[14]	30.4 (6.5)	NA	30.4(6.5)	NA	NA	NA	55.2 (14.2)	NA	55.2(14.2)	NA	NA	NA
Daly *et al. *[15]	35.3 (8.6)	NA	35.3(8.6)	NA	NA	NA	74.9(15.3)	57(9)	75(15)	72(13)	-15(-20 to -11)	<0.001

Data are reported as mean (SD), or median (IQR) unless otherwise indicated. BMI: Body Mass Index. EOT: End Of Treatment. NA: Not Applicable. CI: Confidence Interval.

*Body weight remained unchanged 97(16) Kg, p=0.64

**Δweight (Kg) 2.8 (-0.4 to 6.1), P=0.08.

**Table 2B:** Treatment outcomes of the participants in randomized controlled trials.

**Study ID**	**TIR** **(%)**	**TIR-EOT(%)**	**TIR-control (%)**	**TIR-control EOT(%)**	**ΔTIR(95% CI)**	***p***	**Insulin dose(u/d)**	**Insulin dose -ΕΟΤ(u/d)**	**Insulin dose-control (u/d)**	**Insulin dose-control EOT (u/d)**	**Δinsulin (95% CI)**	***p***
Kudva *et al. *[11]	48 (24)	64(16)	51(21)	52(21)	14(11 to 17)	<0.001	95(47)	87(46)	102(50)	104(56)	-10(-20 to -0)	NA
Borel *et al. *[12]	58.9 (20.1)	76(69-84)	58.9(20.1)	61(55-70)	15(8-22)	<0.001	78.1(35.9)	103.7(76.1-122.8)	78.1(35.9)	78(59.3-95.3)	10 (3.3-30.6)	0.003
Reznik *et al. *[13]	34.4 (21.3)	63(9.4)	38.9(23.2)	36.6(22)	27.4(15 to 39.8)	<0.001	62.8(37.2)	80.6(61.8)	81.8(38.2)	111.7(72.4)	-18.5(-78.2 to 41.2)	0.52
Boughton *et al. *[14]	37.7 (20.5)	52.8(12.5)	37.7(20.5)	37.7(20.5)	15.1(8 to 22.2)	<0.001	0.39* (0.25-0.59)	20.4(9.2-50.3)	0.39* (0.25-0.59)	32.2(12.1-54.4)	NA	0.38
Daly *et al. *[15]	NA	66.3(14.9)	NA	32.3(24.7)	35.3(28 to 42.6)	<0.001	NA	108(73-188)	NA	84(54-129)	NA	NA

Data are reported as mean (SD), or median (IQR) unless otherwise indicated. **BMI**: Body Mass Index. **TIR**: Time In Range. **EOT**: End Of Treatment. **NA**: Not Applicable. **CI**: Confidence Interval.

* Reported as units/Kg/day.

**Table 3:** Baseline characteristics of the participants in non- randomized and real-world trials.

**Study ID**	**AID**	**Duration**	**Sample Size (N)**	**Age (yrs)**	**Diabetes duration (yrs)**	**BMI (Kg/m^2^)**	**HbA1c (mmol/mol)**	**TIR** **(%)**	**Insulin dose** **(units/d)**	**GLP-1RA N(%)**	**SGLT-2 inh N(%)**
Davis *et al. *[17]	Omnipod 5	8 weeks	24	60.6 (8.1)	19(9)	33.5(4.4)	79.2(9.9)	36.6(19.6)	61.5(46.4)	11(46)	6(25)
Davis *et al. *[18]	Omnipod 5	26 weeks	22	61.2(7.7)	18.6(9.4)	33.9(4.4)	79.2(8.8)	36.6(19.6)	64.6(47.2)	9(41)	5(23)
Pasquel *et al. *[19]	Omnipod 5	13 weeks	305	57 (11)	17(11-24)	35 (8)	66.1(14.2)	45 (25)	79.9(50)	168(55)	135(44)
Bhargava *et al. *[20]	Minimed 780G	90 days	95	60.3(10.8)	18.6(8.6)	36(7.4)	62.8(11)	72.2(17.1)	77.4(38.5)	17(18)	20(21)
Breton *et al. *[21]	Control-IQ	12 months	378	NA	20.76(10.3)	NA	NA	69.9(55.1-82.6)	NA	NA	NA

Data are reported as mean (SD), or median (IQR) unless otherwise indicated. AID: Automated Insulin Delivery. BMI: Body Mass Index. TIR: Time In Range. GLP-1RA: Glucagon-Like Peptide 1 Receptor Agonist. SGLT-2inh: Sodium-Glucose Co-Transporter 2 inhibitor. NA: Not Applicable.

**Table 4:** Treatment outcomes of the participants in non-randomized and real-world trials.

**Study ID**	**BMI (Kg/m^2^)**	**BMI-EOT (Kg/m2)**	**ΔBMI (95% CI))**	***p***	**HbA1c (mmol/mol)**	**HbA1c-EOT (mmol/mol)**	**ΔHbA1c (95% CI)**	***p***	**TIR** **(%)**	**TIR-EOT (%)**	**ΔTIR(95% CI)**	***p***	**Insulin dose** **(u/d)**	**Insulin dose-EOT(u/d)**	**Δinsulin** **(95% CI)**	***p***
Davis *et al. *[17]	33.5(4.4)	33.9(4.3)	0.4*(1.3)	0.15	79(10)	65(8)	-14*(8)	<0.0001	36.6(19.6)	58.6(15.9)	21.9*(15.2)	<0.0001	61.5(46.4)	52.6(34)	-8.8(-26.3 to 3.9)	0.07
Davis *et al. *[18]	33.9(4.4)	31.9(3.9)	-0.5*(2.2)	0.51	79(8.7)	62(7.7)	-14.2*(7.7)	<0.0001	36.6(19.6)	61.4(15.2)	22.4*(19.2)	<0.0001	64.6(47.2)	68.7(28.5)	7.4*(20)	0.77
Pasquel *et al. *[19]	34.9 (7,5))	35.1(7.6)	0.3(0.1 to 0.4)	<0.001	66.1(14.2)	57.4(9.8)	-8.7(-10.9 to -7.6)	<0.001	45 (25)	66(17)	20(18 to 22)	<0.001	79.9(50)	57.3(33)	-22.7(-26.7 to-18.7)	<0.001
Bhargava *et al. *[20]	36(7.4)	36.4(7.6)	0.3*(2.4)	0.371	62.8(11)	55.2(7.6)	NA	NA	72.2(17.1)	79.8(11.4)	7.2*(13.8)	<0.0001	77.4(38.5)	91.8(49.	13.9*(28.7)	<0.0001
Breton *et al. *[21]	NA	NA	NA	NA	NA	NA	NA	NA	69.9(55.1-82.6)	78(66.2-86.1)	NA	<0.001	NA	NA	NA	NA

Data are reported as mean (SD), or median (IQR) unless otherwise indicated. **BMI**: Body Mass Index. **TIR**: Time In Range. **NA**: Not Applicable. **EOT**: End Of Treatment. **CI**: Confidence Interval.

*Reported as mean difference (SD).

## LIST OF ABBREVIATIONS

AID: Automated Insulin Delivery
T1DM: Type 1 Diabetes Mellitus
T2DM: Type 2 Diabetes Mellitus
HbA1c: Glycosylated Hemoglobin A1c
CI: Confidence Interval
TIR: Time In Range
CV: Coefficient of Variation
GMI: Glucose Management Index
DKA: Diabetic Keto-Acidosis
IQR: Interquartile Range
GLP-1RA: Glucagon-Like Peptide Receptor Agonist
SGLT-2 inh: Sodium-Glucose Co-Transporter 2 Inhibitors
CGM: Continuous Glucose Monitoring
MDI: Multiple Daily Injections
